# Exercise in Heart Disease

**Published:** 1898-04-16

**Authors:** 


### EXERCISE IN HEART DISEASE.

In dealing with this subject in the Lumleian Lectures Sir R. Douglas Powell speaks of exercise as having been reduced to a system in three grades—massage, resistance exercises, and the graduated walking exercises of Oertel. Massage is useful as a means of helping in the convalescent stage of acute heart affections; of combating the tendency to stagnant circulation in those who are disabled in chronic heart disease; and still more as a means of maintaining the circulation in those who, being bed-ridden or sofa-ridden from any other cause, tend on that account to impairment of heart nutrition and its consequences. In regard to acute heart disease, however, massage is not to be advised. These acute affections mostly occur in young persons whose hearts are not disposed to degeneration, and whose muscles recover with an energy that has to be restrained rather than encouraged. So long as there is any activity or softness about the valve lesion we must not excite the heart to increased action, and so long as there is any daily rise of temperature massage treatment should be discouraged. In regard to "resistance exercises," Sir R. Douglas Powell speaks with very great caution, and he does not accept all the *dicta* of the Schott school. "It seems to me," he says, "that the carefully graduated and observed exercises of Schott and Oertel may be regarded as a counsel of perfection to be advised for those who can afford them as a preliminary to the return to that measure of active life of which their heart condition admits, and as a guide indicative of what that measure will be, and by what degrees of ordinary exercise it may be arrived at." Again, resistance exercises are especially adapted for the initial treatment of flabby, irritable, "stuffy" hearts, applying that term to cases of fatty infiltration and impaired metabolism in people of venous plethora. In chlorosis with dilated heart, after a week or two of complete rest, the Schott treatment is valuable, if combined with a dry, bracing climate, and a chalybeate. In commencing failure of heart in chronic valve lesions the treatment may also be employed with a more or less complete cessation from all other exercises, and similarly after such cases have been restored up to a certain point by digitalis. Cases of atheromatous changes in the coronary vessels, characterised by a certain degree of plethora, and by breathlessness attended by cardiac pain on arriving at a certain stage of walking exercise, may also be cautiously treated by the same method. On the other hand, there are cases in which this treatment should not be employed. All cases of acute endocarditis, while there is any trace of activity of lesion, are still more unsuitable for this treatment than for massage. Cases of advanced cardiovascular changes of the nature of sclerosis, and particularly when associated with granular kidneys, are absolutely unsuited. In cases of introspective people with neurotic hearts the treatment is best avoided, while the numberless cases of imaginary weak hearts that would naturally flock to specified "cures" really require a diagnosis and a better occupation than that of indoor gymnastics. Neither in tachycardial cases nor in exophthalmic goitre do the exercises appear to be successful. Oertel's treatment by graduated walking exercise, and a dry non-fatty, non-starchy diet, as fully described in *Ziemssen's Cyclopædia,* may be regarded as a sequel to the Schott treatment. One great advantage is that it is undertaken in the open air, for there is little doubt that the discomfort which sometimes ensues from massage and passive exercises is often to be attributed to want of the abundant air required for the increased oxidation produced.

